# Photoaggravation of Hair Aging

**DOI:** 10.4103/0974-7753.58551

**Published:** 2009

**Authors:** Won-Soo Lee

**Affiliations:** Department of Dermatology, Institute of Hair and Cosmetic Medicine, Yonsei University Wonju College of Medicine, Wonju, Kangwon-Do, Republic of Korea

**Keywords:** Hair aging, integral hair lipid, melanin, photoaging, photoaggravation, protein, ultraviolet light

## Abstract

Photoaggravation of hair aging includes various chemical and physical changes in fiber properties which lead to an increase in fiber porosity, loss of mechanical strength and an increase in surface roughness. These changes come from lipid oxidation, disulfide bond cleavage, tryptophan degradation and cysteic acid formation. Hair exposed to sunlight is claimed to be more brittle, stiffer and drier than before irradiation and exhibits a reduced water-absorption capacity. Hair pigments function to provide photochemical protection to hair proteins. Hair pigments accomplish this protection by absorbing and filtering the impinging radiation and subsequently dissipating this energy as heat. However, in the process of protecting the hair proteins from light, the pigments are degraded or bleached. Dark hair is more resistant to photodegradation than light hair, because of the higher photostability of eumelanin compared to pheomelanin. Integral lipids of hair fibers are degraded by ultraviolet light, as well as by visible light, helping to explain the weakening of the cell membrane complex exposed to light radiation.

## INTRODUCTION

Hair aging comprises of degradation of the hair shaft, which involves progressive degeneration of the hair fiber from the root to the tip. Human hair is constantly subjected to repeated environmental assaults, commonly termed *weathering*, which is aggravated by various extrinsic damages. Extrinsic factors which cause weathering include sunlight, water, dust, friction, hair combing, cosmetic hair treatments such as hair dyeing or permanent waving. These factors cause extrinsic hair shaft aging in addition to natural intrinsic hair shaft aging.[1,2] Among the natural aggressors, sunlight in particular, ultraviolet (UV) rays play an important role in hair aging. In light-induced aging, the hair becomes paler in color, and changes that impair the softness and shine of hair occur in the surface condition. Hence light modifies the cosmetic properties of hair. Hair fibers therefore need to be protected from light.[3] Exposure to light radiation is known to cause many undesirable effects. Although it is not immediately perceived, UV damage to hair fibers plays an integral role in the overall aspects of hair damage.[4]

Photochemical degradation of hair results in attack on both hair protein and hair pigment. Changes in hair fibers induced by UV light are largely composed of physical and chemical changes. As for physical changes, dryness, reduced strength, rough surface texture, loss of color, decreased luster, stiffness and brittleness may occur. Chemical changes can occur in hair proteins, lipids and hair pigments.[5] Lipid oxidation, disulfide bond cleavage, tryptophan degradation and cysteic acid formation lead to an increase in fiber porosity, loss of mechanical strength and an increase in surface roughness.[6-8] The photochemical effects on hair color are strongly dependent on the presence of melanins and chromophores in the hair.[9] Pheomelanin is more sensitive than eumelanin.[10] Hair pigments function to provide some photochemical protection to hair proteins, especially at lower wavelengths. Hair pigments accomplish this protection by absorbing and filtering the impinging radiation and subsequently dissipating this energy as heat. However, in the process of protecting the hair proteins from light, the pigments are degraded or bleached.[10] UV light and oxygen affect not only the melanin but also the amino acids and fatty acids in the hair and on the cuticle.

## VISIBLE CHANGE IN PHOTOAGING HAIR

The definite evidences of natural aging are changes in the visual properties of hair, viz., loss of color, shine; and surface properties, viz., wettability and softness.[11] Images obtained by transmission electron microscopy of hairs exposed to sunlight show that melanin pigments have been loosened from their envelope and that some have vanished. This explains the loss of hair color. The microfibrillar structure appears unaltered. However, cuticle cells are missing or detached, the endocuticle is affected and intercellular adhesion seems to be impaired. Morphological changes undergone by light-exposed cuticle explain the rise in the friction coefficient of a hair, the increased fragility of the cuticle and increased porosity.[12] The hair therefore becomes more vulnerable to mechanical or chemical treatments. Degradation of the cortex was shown by loss in mechanical resistance of light-exposed hair.[13] Both the tensile strength and the elongation at break are diminished in irradiated hair.[13]

Long-term ultraviolet exposure causes severe chemical degradation to the hair proteins. As previously indicated, the damage is so extensive that structural differentiation is diminished. This physicochemical degradation usually occurs at a higher level in the hair fiber periphery, with a gradient to a lower level of oxidative damage deeper inside the fiber. Such damage leads to unusual fracture patterns during extension. This includes the breakdown of disulfide bridges within structural units and the establishment of new intramolecular and intermolecular cross-links via reaction of carbonyl groups with amino groups within and between structural units, thereby decreasing structural integrity. These reactions most likely lead to a gradual increase in brittleness and a gradual loss of structural differentiation.[14] Subsequent exposure to aqueous alkaline solution or to peroxide solution leads to rapid dissolution of the affected areas even during short-term exposure to peroxide solutions (bleaching solutions). Longer exposure of these UV-radiated fibers to oxidizing solutions leads to dissolution/elimination of scale differentiation and dissolution of the melanin granules.[14] Robbins and Kelly have analyzed amino acids of both proximal and distal ends of human hair and have shown significantly more cysteic acid in distal ends.[15] They attribute this change to weathering actions, specifically to ultraviolet radiation. This same study also found significant changes in tyrosine and histidine similar to the weathering effects in wool fiber. Decrease was also reported in the lysine content in this study on hair weathering.[15]

The most obvious effect of the photoaging of human hair is hair lightening, an effect that is accelerated by moisture.[16-18] The extent of this photochemically induced color change is dependent on the nature of the hair pigment and is understood to involve an oxidative attack on the eumelanin or pheomelanin melanosomes.[19] Black hair is more photostable than blond hair. Black hair seems well protected against UV light, and light brown hair is obviously damaged by a wide range of natural sunlight. The protective action of melanin granules is limited to the melanin-rich cortex of black hair, which shows only a slight modification of fiber proteins under irradiation.

## ALTERATION OF HAIR PROTEIN IN PHOTOAGING HAIR

The amino acids of the cuticle are altered to a greater extent by sunlight than the amino acids of the cortex because the outer layers of the fiber receive higher intensities of radiation.[14] The following amino acids have been shown to be degraded by light radiation on wool fiber:[20,21] Cystine and methionine (the sulfur containing amino acids); the aromatic and ring amino acids phenylalanine, tryptophan, histidine and proline; and the aliphatic amino acid leucine. Hoting *et al*.[10] have shown that the proteins of the cuticle are degraded by UVB and UVA, but much less by visible (VIS) light; and that cystine, proline and valine are degraded more in light brown hair than in black hair. In other words, the photoprotective effect is much better in dark hair than in light hair. Oxidation at the peptide backbone carbon has been shown to occur from UV exposure both in wool[22] and in hair,[23] producing carbonyl groups (alpha keto acid/amide) and amide groups. The formation of carbonyl groups is favored in the dry state reaction more than in the wet state reaction.

It has been suggested that ubiquitin could be an indicator of hair damage.[24] Stable protein portions in normal hair are transformed to labile protein, the internally formed soluble protein which is accumulated in damaged hair shaft. The major components of the labile protein are ubiquitins. Labile protein must be associated with hair damage and could possibly provide marker for hair photodamage.[5] S100A3, a unique protein among all members of the calcium-binding S100 family, is specifically expressed at the inner endocuticle of human hair fibers. Upon hair damage, S100A3 is released from hair fibers and possibly destabilizes the hair tissue architecture.[25] S100A3 protein is specifically expressed in the cuticular cells of human hair shaft[26] and in the cuticle of murine pelage follicle.[27] In human hair fiber, the protein is segregated in the endocuticle of cuticular cells and in the matrix that surrounds macrofibril bundles in cortical cells.[28] Thus, S100A3 could possibly provide structural integrity to the hair fiber and must be associated with hair damage.[25] S100A3 is released from the cuticle of hair fibers during washing and rinsing, especially from chemically treated or UVB-irradiated fiber.[25]

When UVB radiation hits hair, it has to penetrate a layer of absorbing molecules of about 5 μm thickness, the intensity decreasing exponentially.[29] Considering that intact hair cuticles are 6 ~ 10 layers, each with a thickness of 0.3~;0.5 μm, UVB seems mainly to affect the cuticles. This suggests that hair damage by UVB irradiation is mainly confined to the superficial layers of the hair shaft, the cuticle layers. UVA irradiation can penetrate deeply into the cortex, and so photochemical changes of cuticle and cortex together, may appear greater after UVA irradiation.[5] On the other hand, UVB causes severe morphological damage, confined to the hair cuticles because of its restricted depth of penetration.[5] Morphological evaluation showed relatively more destructive cuticular changes after UVB irradiation than after UVA, while disruptions of the intercellular lipid layer show similar results between UVA and UVB irradiation. However, in labile protein analysis,[24] damaged labile hair proteins were much more observed after UVA irradiation than after UVB irradiation.[5]

## PHOTOLIGHTENING OF HAIR MELANIN

Black hair contains more or less 99% eumelanin and 1% pheomelanin; dark brown and blond hairs, 95% and 5%; and red hair, 67% and 33%.[30] Generalizing, dark hairs have more melanin and more photosensitive amino acids than light hairs. Melanin can attribute photoprotection to hair protein, but only in the cortex. As dark hairs have more photosensible proteins than light hairs, they can show a greater protein loss than light hair in the cuticle region. In the cortex, even though dark hairs have more photosensible proteins than light hairs, they also have more melanin to absorb the UV radiation.[31]

Red hair was found to lighten to a similar extent by irradiation from both UV and VIS light. On the other hand, under the same irradiating conditions, blond hair was lightened by VIS light but was not lightened by UV light until it was washed after irradiation.[32] These different photolightening behaviors of red and blond hair are supposed to be due to differences in their melanin compositions. The dominant type of melanin in red hair is pheomelanin; while blonde hair contains both eumelanin and pheomelanin, with mainly eumelanin. It has been proven that chemically intact melanin in red hair is considerably more photolabile to UV light than to VIS light. Also, it is much more easily decomposed by UV light than the melanin granules in blond hair, although they are both similarly decomposed by VIS light. This indicates that pheomelanin is far more sensitive to UV light than eumelanin, while these two types of melanin are similarly sensitive to VIS light.[33]

## PHOTOCHEMICAL MECHANISM OF PROTEIN CHANGE

Light radiation causes degradation of cystine, but the exact mechanism is not well known. Photodegradation of cystine occurs through the C—S fission pathway and is different from the chemical oxidation of cystine that proceeds mainly via the S-S fission route.[14] Savige and Maclaren[34] also suggest the C—S fission route as the preferred route for photochemical degradation of cystine and of other pure disulfides. Changes in mechanical properties of irradiated human hair correlate with the photodamage of the hair proteins.[16,35] It was shown that the C—S bonds of cystine are cleaved upon UV radiation.[16] With long-term irradiation, there is a progressive decrease in the total recovery of amino acids after hydrolysis, except for alanine, glycine and arginine. However, there is no clear pattern of protein degradation.[36] For the S-S fission route, the main end product is sulfonic acid. For the C—S fission route, the main products are the S sulfonic acid and sulfonic acid.[34] However, ultimately, S sulfonic acid is degraded by light to sulfonic acid.[34] These results suggest that the mechanism for the radiation-induced degradation of cystine occurs through the C—S fission pathway and is different from the chemical oxidation of cystine, which proceeds mainly via the S-S fission route. Differentiating the effects of UVA and UVB radiations on hair structure, Ruetsch *et al*.[8] also studied hair photodamage under conditions of prolonged exposure to UV radiation, and hair was found to undergo substantial changes, both chemical and morphological. Photodegradation of hair proteins was more pronounced in unpigmented/blonde hair, the highest level of photodegradation occurring in the cuticular region, where cystine is present at its highest concentration. Prolonged UV irradiation produced the thinning and the fusion of cuticular cells; the proposed theory is that the outer proteic layers would photodegrade to smaller, lower-'molecular weight' peptides, which would then diffuse into the hair structure when enough humidity would allow for the fiber's swelling.[37]

## MECHANISM OF PHOTOLIGHTENING

The photochemical degradation by VIS light of eumelanin is relatively small and can be shown quantitatively (gravimetry) and qualitatively (color measurements, IR spectroscopy).[10] On the other hand, a mixture of pheomelanin and eumelanin, the pigment of light brown hair, appears to be affected by all segments of sunlight, particularly by UVA and VIS light. The irradiation brings about drastic degradation of the granules and substantial changes in the melanin polymer.[10] IR investigation of the melanin of irradiated light brown hair suggests extensive destruction of the quinine system, which according to Crippa[19] is essential for the photoprotective effect of melanin. In the case of eumelanin, the dihydroxyindole moieties are prone to irradiation.[10] The higher photostability of the eumelanin, particularly the limited degradation of the quinine structure, suggests that eumelanin has a better photoprotective effect on hair than pheomelanin.[10] Eumelanin ring opening results from free radical reaction. Slawinska and Slawinski[38] have suggested that photochemical degradation of melanin occurs through similar peroxide intermediate. The first step in the photochemical degradation of the eumelanin chromophore probably involves excitation to a radical anion and then attack by the oxygen radical anion on the 0 quinone grouping. Ring opening of the six-membered ring indolequinone species then follows. A related scheme may be involved for the photochemical degradation of pheomelanins. However, Arakindakshan *et al*.[39] have shown that pheomelanins are more easily induced to an excited state than the brown black eumelanins. Hoting *et al*.[10] have shown that the pigment of light brown hair is affected by UVA, UVB and visible light, but eumelanins are much light and stable and provide a greater photoprotective effect.

Although there is a general agreement in the literature that melanin gives photochemical protection to hair, many contradictions are found about the photostability of pheomelanins and eumelanins. Hoting *et al*.[40] state that dark hair is more resistant to photodegradation than light hair, because of the higher photostability of eumelanin compared to pheomelanin. On the other hand, Wolfram and Albrecht[18] claim that pheomelanin is less vulnerable to photodegradation than eumelanin. But hair damages caused by UV exposure are not related only to the melanin type of each hair. Most likely, the smallest protein loss observed for black hair is associated to the greater amount of melanin in this hair as compared to others. In other words, a hair with more pigment granules will show a smaller protein loss when exposed to the ultraviolet range that is absorbed by both hair pigments and hair proteins. Also, protein loss is observed after only 56 hours of UVB radiation exposure, when probably only cuticle proteins are damaged, and this is also a clue that the photoprotective effect of melanin is related to the total amount of melanin in the hair and not just with the melanin type.[31]

## OXIDATIVE STRESS IN PHOTOAGING HAIR

UV radiation induces the formation of oxy radicals such as superoxide and hydroxyl. These species have one unpaired electron in an outer orbital giving them a very powerful aptitude to react, especially with molecules having a double bond in their structure, such as unsaturated lipids.[41] Chemically, these changes are thought to be caused by 'UV light'-induced oxidation of the sulfur-containing molecules within the hair shaft. Melanin has an intrinsic electron spin resonance (ESR) signal that increases significantly when irradiated with UV and VIS light. In the presence of oxygen, superoxide is produced, which dismutates to hydrogen peroxide. This leads to the formation of hydroxyl radicals in the presence of trace amounts of metal ions. Oxidation of the amide carbon of polypeptide chains also occurs, producing carbonyl groups.[42] This process has been studied extensively in wool, where it is known as photoyellowing.[43,44] The photodegradation of cystine is the most conspicuous alteration on the amino acid residue of the whole fiber, to which additional consequences for the hair are attributable: Loss of fiber strength; and water infiltration, which subsequently creates favorable conditions for further photo-oxidative reactions by the dissolved oxygen.[36] Robbins and Bahl[23] have examined the effects of sunlight and ultraviolet radiation on disulfide bond in hair via electron spectroscopy for chemical analysis.[23] Both UVA and UVB radiations were shown to oxidize sulfur in hair. The primary oxidation occurs closer to the hair fiber surface, producing a steep gradient of oxidized to less oxidized hair from the outer circumference of the hair to the fiber core.

## CHANGE OF INTEGRAL HAIR LIPID IN PHOTOAGING HAIR

Cuticle cells are covered with a thin integral lipid (IL) layer covalently bonded to hair proteins.[45] The chemical composition of IL is different from that of epidermal or sebaceous lipids.[46] The lipid content of human hair takes up to 0.7%~1.3% in total components of hair. The IL in hair is the only continuous structure that plays a key role in maintenance of hair integrity, including hydrophobicity and stiffness.[47] Cuticle cells in hair are abundant in fatty acids unlike the keratinized area of epidermis or sebaceous gland, and about 40% of such fatty acids are composed of 18 methyleicosanoic acid (18 MEA) and known to be bound to proteins by ester or thioester bond in living or keratinized cells.[48,49] There have been studies on the IL between hair cuticles in hair; it has been known to be present as a cell membrane complex (CMC) composed of a δ layer, core structure relatively light stained, covered with two β layers in sandwich form.[47] Using the example of cholesterol and free fatty acids, it could be demonstrated that the visible range of sunlight destroys IL to a considerably higher extent than exposure to UVB and UVA ranges. The photochemical destruction of lipids is largely retarded by the pigment eumelanin.[50] It is difficult to observe the IL with the conventional staining method using OsO_4_ or RuO_4_. OsO_4_ cannot reveal lipid component in tissue. On the other hand, RuO_4_, which is routinely used to reveal epidermal lipids, causes severe hair damage. So a new fixative (Lee's fixative) to minimize hair injury was designed to observe the IL in hair.[46] With this technique, it is possible to observe UV-induced CMC lipid damage directly.

According to recent investigations on human hair, a correlation exists between the amount of IL and the moisture content in the hair.[51] A reduction in the amount of lipids by solvent extraction[52] and oxidative damage during chlorine bleaching of wool[53] or during permanent waving of hair[54] favor the diffusion of foreign materials into the fiber.[55] Hoting *et al*.[50] have shown that the CMC lipids of hair fibers are degraded more by visible light but also by UVA and by UVB light, helping to explain the weakening of the CMC and the multiple-step fractures observed in hair exposed to light radiation.

Irradiation with sunlight degrades the IL in blond hair more than that in black hair. UVB and UVA destroy in blond hair approximately 25% of the cholesterol. Even visible (VIS) light and global radiation degrade 60% and more of the initial cholesterol content. In contrast, the cholesterol fraction from black hair is not significantly altered by UVB, UVA and VIS light; in contrast with blond hair, global radiation leads only to a lower extent (24%) of photo-oxidation of cholesterol in black hair.[50] The photochemical degradation of free fatty acid (FFA) occurs in blond and black hair by the influence of UVB and UVA to comparable degrees. UVB irradiation reduces the FFA amount by approximately 40%; and UVA irradiation, by approximately 20%. Differences as a function of the type of pigmentation can be detected for hair irradiated with VIS light. The FFA fraction from blond hair was reduced by only 23%. With the exception of the FFA fraction from black hair, IR irradiation does not show a significant degradation of lipids. Global irradiation causes in blond hair a degradation of fatty acids of 33%; and in black hair, of 42%.[50] The lower photo-oxidative degradation in black hair suggests that eumelanin is responsible for this effect. It protects the IL mainly from the photochemical influence of the visible range of sunlight. The protective function of melanin against photodegradation also applies to the UVB and UVA ranges.

## CONCLUSION

Photodamaged hair shows dryness, reduced strength, rough surface texture, loss of color, decreased luster, stiffness and brittleness. Ultraviolet light and oxygen affect not only the melanins but also the amino acids and lipids in the hair and on the cuticle. Several amino acids of hair absorb light in this region, and these amino acids are the ones most subjected to degradation by light. The photochemical effects on hair color are strongly dependent on the presence of melanins and chromophores in the hair. Eumelanin is much light and stable and provides a better photoprotective effect on hair than pheomelanin. Melanin can attribute photoprotection to hair protein, but only in the cortex. As dark hairs have more photosensible proteins than light hairs, they can show a greater protein loss than light hairs. In the cortex, even though dark hairs have more photosensible proteins than light hairs, they also have more melanin to absorb the ultraviolet radiation. Integral lipids of hair fibers are degraded by ultraviolet light as well as by visible light, cause weakening of the cell membrane complex exposed to light radiation.
